# Characterization and Antioxidant Activity of Collagen, Gelatin, and the Derived Peptides from Yellowfin Tuna (*Thunnus albacares*) Skin

**DOI:** 10.3390/md18020098

**Published:** 2020-01-31

**Authors:** Mala Nurilmala, Hanifah Husein Hizbullah, Euis Karnia, Eni Kusumaningtyas, Yoshihiro Ochiai

**Affiliations:** 1 Department of Aquatic Product Technology, Faculty of Fisheries and Marine Science, Bogor Agricultural University (IPB University), Bogor 16680, Indonesia; hhanifaa@gmail.com (H.H.H.); euiskarniaa@gmail.com (E.K.); 2 Indonesian Research Centre for Veterinary Science, Bogor 16114, Indonesia; enikusuma@yahoo.com; 3 Graduate School of Agricultural Science, Tohoku University, Sendai 980-8572, Japan; yochiai@tohoku.ac.jp

**Keywords:** antioxidant, collagen, fractionation, gelatin, hydrolysate, tuna skin

## Abstract

Skin waste from tuna processing needs to be utilized, such as extraction of its collagen and gelatin. Their functional properties can be improved by enzymatic hydrolysis for conversion to peptides. Thus, the research objectives were to examine the characteristics and antioxidant activity of collagen, gelatin, and the derived peptide from yellowfin tuna skin. Collagen was extracted using 0.75 M acetic acid at 4 °C, while gelatin was prepared using 0.25% citric acid and extracted at 65 °C. Hydrolysis was carried out with 2% Alcalase, followed by fractionation with a molecular weight cut off sieve for both collagen and gelatin. Collagen yield was 22.6% with pH value of 6.63 and whiteness of 96.7%. Gelatin yield was 20.0% with pH value of 4.94 and whiteness of 51.0%. Hydrolysis for three hours resulted in 52.7% and 45.2% degree of hydrolysis for collagen and gelatin, respectively. The molecular weights of collagen peptides ranged from 2.94 to 11.93 kDa, while those of gelatin peptides ranged from 3.54 to 16,620 kDa. Antioxidant activities of these peptides were higher than those before hydrolysis. The high antioxidant activity (IC_50_) of collagen peptides were found in <3, 3–10, and 10–30 kDa fractions as well as in the gelatin peptides.

## 1. Introduction

Tuna is one of the marine biotas that has high economical value. The global total annual catch of tuna species was around 7.5 million tons in 2018 based on The State of World Fisheries and Aquaculture by Food and Agriculture Organization (FAO) [1]. The United States of America market imported nearly 42,400 tons of fresh and frozen tuna during January to September 2016, at a value of US $439.2 million. Among these imports, frozen tuna fillets took a 51% share, followed by whole, dressed, and fresh/chilled tuna (43%). Demands for frozen tuna loins in Japan have been increasing about 3% every year. Thailand, which bought 13% more cooked loins at 26,000 tons for reprocessing, is the largest importer of tuna raw material in Asia. China, Vietnam, and Indonesia are the main suppliers [2]. Fishermen of nearly 80 nations harvest tunas from the world oceans. Yellowfin tuna (*Thunnus albacares*) is the second most important species of tuna, which accounts for about 30% of the global catch [3]. Tunas are commonly produced for canning and freezing. Frozen products are usually in the form of whole fish or loins. The freshness and quality of tuna meat is usually evaluated based on the oxidation extent of the muscle pigment, myoglobin [4]. The frozen tuna products could leave bones and skin wastes. The proportion of tuna skin is about 5–8% of the total fish weight. Tuna bones and skins are generally used only to feed cattle with reduced selling price. For value addition, the skin waste from industrial tuna loins as well as fillets can be utilized as the materials for collagen and gelatin [5,6,7]. However, a large amount of tuna skin has remained underutilized, despite the fact that it can be an excellent source of collagen and gelatin, provided that they are demonstrated to be of high quality. The bottleneck for the utilization of tuna skin is the high fat content.

Commercial collagen and gelatin are generally produced from the skins and bones of pigs and cows. However, their usage raises religious issues, because Hinduism forbids consumption of cows, while Islam and Judaism forbid that of pigs. In addition, there are associated health concerns such as bovine spongiform encephalopathy (BSE), transmissible spongiform encephalopathy (TSE), and foot and mouth disease (FMD). Therefore, fishery by-products such as skin, bone, scale, and swim bladder could be alternatives for collagen and gelatin production [8,9,10]. The yellowfin tuna skin waste could be an alternative raw material for making safe and preferable collagen and gelatin.

Collagen is a connective tissue protein found in skin, teeth, and bones. Collagen can be converted to gelatin by high temperature treatment. Both collagen and gelatin—which contain glycine, proline, and hydroxyproline as the major amino acids—have significant potential as the initial substrates to produce bioactive peptides [11,12]. The biological properties of the derived peptides are largely dependent on the structure and molecular weight. Enzymatic hydrolysis is an effective method for obtaining bioactive peptides [13]. Recently, the bioactive peptide with antioxidant properties from enzymatically hydrolyzed proteins is a topic of great interest in the pharmaceutical and food processing industries [14]. Previous researchers reported that Alcalase showed the most effective among five kinds of proteases to hydrolyze gelatin from skipjack *Katsuwonus pelamis* scales, resulting in strong antioxidant activities of the peptides obtained [8]. Antioxidant supplementation can maintain normal skin cell function by preventing free radical accumulation [15]. Superior antioxidant activity of peptides prevents oxidative stress which is the main cause of aging. Biomolecules with strong antioxidant capacity are widely used in anti-aging research [16]. Under these backgrounds, this study aimed to investigate the characteristics of tuna skin collagen and gelatin, and the antioxidant activity of their hydrolysates. 

## 2. Results and Discussion

### 2.1. Physicochemical Characteristics

Physicochemical characteristics of collagen and gelatin from yellowfin tuna are shown in Table 1. The yields of collagen and gelatin clearly show the efficiency level of the extraction process. In this study, the yield of collagen from the tuna skin was higher than those of sail fish (*Istiophorus platypterus*, 5.76%) [17], ocellate puffer fish (*Takifugu rubripes*, 10.7%) [18], bighead carp (*Hypophthalmichthys nobilis*, 17.50%) [19], redbelly yellowtail fusilier (*Caesio cuning*, 18.4%) [20], and balloonfish (*Diodon holocanthus*, 4%) [21]. In addition, the gelatin yield in this study (19.97%) was quite high when compared with that of skipjack tuna (*K. pelamis)* skin (11.3%) [22], emperor fish (*Lethrinus* sp.) skin (4.8%) [23], and *K. pelamis* bone (6.37%) [24]. The main structures of the skin inner layers and different harvesting ages could lead to differences in the yield [25].

pH is an important factor for the quality of collagen and gelatin. The pH value of tuna skin collagen preparation was 6.63, which was acceptable in accordance with Indonesian National Standard for collagen pH range, which is 6.5–8 [26]. The pH value of the gelatin was also acceptable in accordance with the Gelatin Manufacturers Institute of America standard as Type A, where the optimal pH is in the range of 3.8–5.5 for the food standard and 4.5–5.5 for the pharmaceutical one [27]. The resulting gelatin was also found to belong to Type A, as it was prepared by an acidic process. The pH value is influenced by the type of material used in the pretreatment with acid or base and the neutralization step [22]. It is known that optimal pH value can make it easier to apply the material to the product. Neutral pH facilitates the interaction of proteins with water molecules, thereby improving the solubility [28].

Color does not affect the functional ability of the product. However, it remains an important factor since consumers would prefer products in brighter colors [22]. The whiteness degree of tuna collagen was 96.69%, higher than those of white snapper collagen (61.33–65.41%) [29] and skate collagen (88.4%) [30]. In this study, it was shown that the resulting L* value (lightness) and whiteness of the gelatin gave lower values compared with those of the original collagen. However, the L* value was higher compared to that of the gelatin from giant catfish (*Pangasius gigas*) skin (20.43) [31]. The a* value (redness) increased about two fold by converting the collagen to the gelatin, demonstrating that the redness was enhanced in the gelatin. On the other hand, the b* value (yellowness) increased nearly tenfold by the conversion, indicating that the yellowness was intensified in the gelatin. Usually, the color of commercial gelatin is pale yellow to dark amber [32]. Darker gelatin is commonly due to inorganic contaminants as well as proteins which are not removed during the extraction process. The color of gelatin is greatly dependent on fish species or the original color of fish skin, as well as conditions of extraction and drying [33].

### 2.2. Molecular Size and Hydrolysis Degree

**Figure 1:**
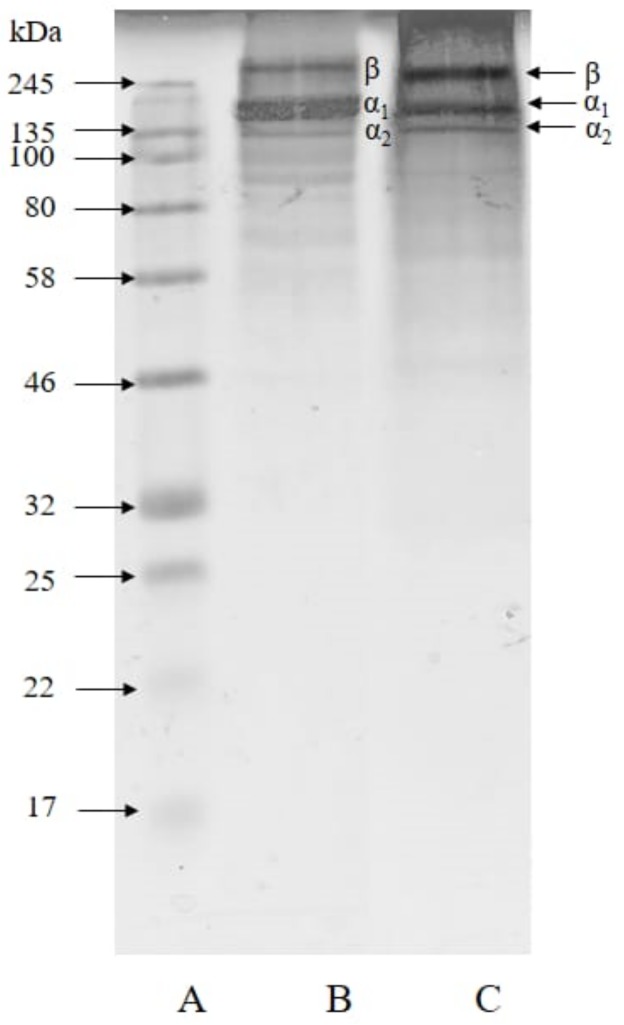
Sodium dodecyl sulphate-polyacrylamide gel electrophoresis (SDS-PAGE) patterns of tuna skin collagen and gelatin. A, markers; B, collagen; C, gelatin.

Molecular size is an important parameter for determining the properties of collagen, gelatin, and the derived hydrolysates. Their molecular weights were estimated by sodium dodecyl sulphate-polyacrylamide gel electrophoresis (SDS-PAGE) [34,35] as shown in Figure 1. 

The tuna skin collagen and gelatin showed similar patterns in that both consisted of β, α1, and α2 subunit bands. They are classified as type I collagen. The molecular weight of collagen was estimated to be 277.17 kDa. Type I collagen consists of α_1_ (±120 kDa) and α_2_ (±110 kDa) chains, and β (±200 kDa) chain [36,37]. The molecular weight of the β component in the gelatin was estimated to be 226.65 kDa, and those of the α_1_ and the α_2_ components were 153.85 kDa and 134.54 kDa, respectively. Our previous study showed the yellowfin tuna skin gelatin extracted at 65 °C consisted of β component of 250 kDa, α1 of 129.67 kDa, and α_2_ of 116.36 kDa [4]. Tuna gelatin has high molecular weight bands judging from abundant β components [13]. The results showed that the molecular weight of yellowfin tuna skin gelatin had a similar molecular size range to that of tilapia scale gelatin *Oreochromis* spp. (34–260 kDa) [38].

Collagen and gelatin were then hydrolyzed with the proteolytic enzyme Alcalase at a ratio of 2% (*v/v*) in order to obtain the hydrolysates. The hydrolysis degree (HD) of these preparations is shown in Table 2. The HD value, generally used as a parameter of proteolysis and the indicator of protein hydrolysate ratio, is the ratio of the reduced peptide bonds during hydrolysis to the total amount of the bonds in the original protein. The HD value may affect the functional properties of the hydrolysate [39]. Alcalase is a proteolytic enzyme of relatively high activity under moderate pH conditions compared with whose which are active at neutral and acidic pH [40].

**Figure 2:**
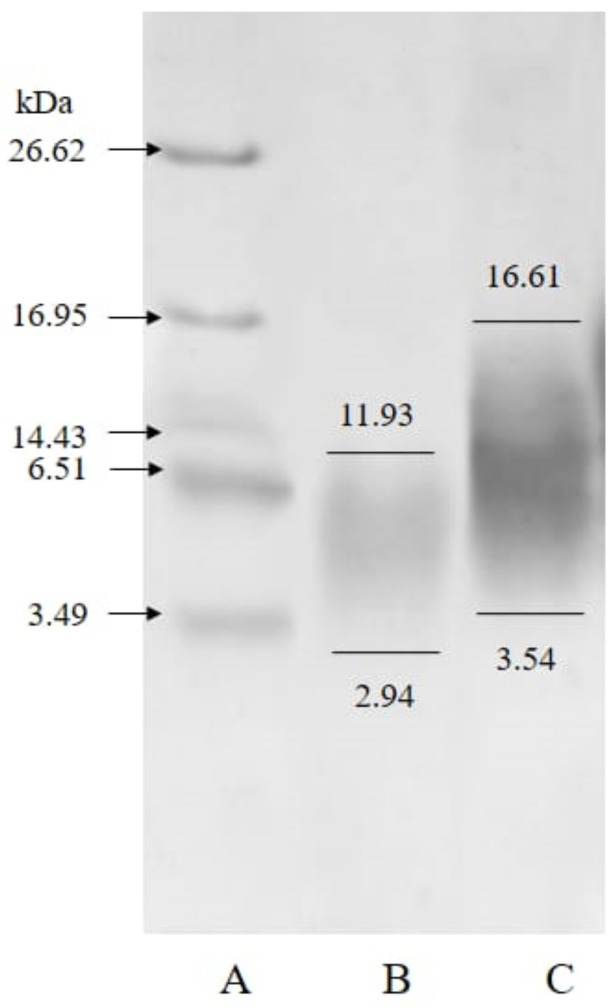
SDS-PAGE patterns of collagen and gelatin hydrolysate. A, markers; B, collagen hydrolysate; C, gelatin hydrolysate.

The molecular weight of collagen hydrolysate as estimated by SDS-PAGE was in the range of 2.94–11.93 kDa as shown in Figure 2. Generally, bioactive peptides of high antioxidant activity have a molecular size of less than 10 kDa [41]. The gelatin hydrolysate in this study was found to fall in the range of 3.54–16.61 kDa. Guerard et al. reported that hydrolyzed gelatin prepared from tuna waste produced peptides of about 5–25 kDa [42]. 

### 2.3. Amino Acid Composition

It is known that collagen and its thermal derivative, gelatin, are rich in amino acids such as glycine and proline. The amino acid compositions of collagen and gelatin from the yellowfin tuna skin and its hydrolysates are shown in Table 3. The composition and sequence of amino acids in gelatin depend on the species of materials, but the major ones are glycine and proline irrespective of the species so far examined [43]. The length of polypeptide chains is closely correlated with the viscosity of gelatin. The longer the polypeptide chain, the higher the gelatin viscosity value [33]. In addition, amino acid composition affects gel strength and the melting point of gelatin [44]. In addition to physical properties, amino acid composition also affects the functional properties of collagen, gelatin, and the hydrolysates, especially the antioxidant activity.

The antioxidant properties of peptides are closely related to amino acid composition, structure, and hydrophobic properties [13]. The amino acid composition of the hydrolysates was very similar to that of the parent proteins, which were rich in glycine, alanine, proline, aspartic acid, and glutamic acid. High glycine and proline contents make the antioxidant activity of fish skin gelatin higher than that of meat protein [45]. Alcalase is able to hydrolyze the bonds of aliphatic or aromatic amino acid peptides such as leucine, phenylalanine, and tyrosine [46]. Table 3 shows that the hydrolysis process lowered the amino acid levels of collagen, by maintaining the amino acid composition. The arginine content, which generally reflects the antioxidant activity, was retained through hydrolysis [47].

### 2.4. Antioxidant Activity

The antioxidant activity was measured by a 2,2′-azino-bis (3-ethylbenzothiazoline-6-sulphonic acid) (ABTS) method as well as a 2,2-diphenyl-1-picrylhydrazyl (DPPH) method in order to examine how much the hydrolysis and fractionation process influenced the antioxidant activities. The ABTS radical is a radical with a nitrogen center that has the coloration of blue/green, when reduced by antioxidant compounds. It will turn into a non-radical form, from colored to colorless, whereas DPPH radicals are violet-colored free radicals that require electron transfer from antioxidants to transform into a colorless non-radical solution. The antioxidant activities of the collagen, gelatin, and the hydrolysates are shown in Table 4.

Antioxidants are the molecules capable of quenching reactive oxygen species as well as inhibiting the oxidation of molecules that can produce free radicals. Antioxidants are electron-donating compounds (electron donors) or reductants [48]. Antioxidant activity is associated with the progress of reductions, which have been confirmed to be terminators of chain reactions caused by free radicals [36]. Free radicals are the major factors that can accelerate glycation [49]. They can also cause oxidative stress and thus various diseases [50]. The antioxidant activities were increased through hydrolysis. 

**Figure 3:**
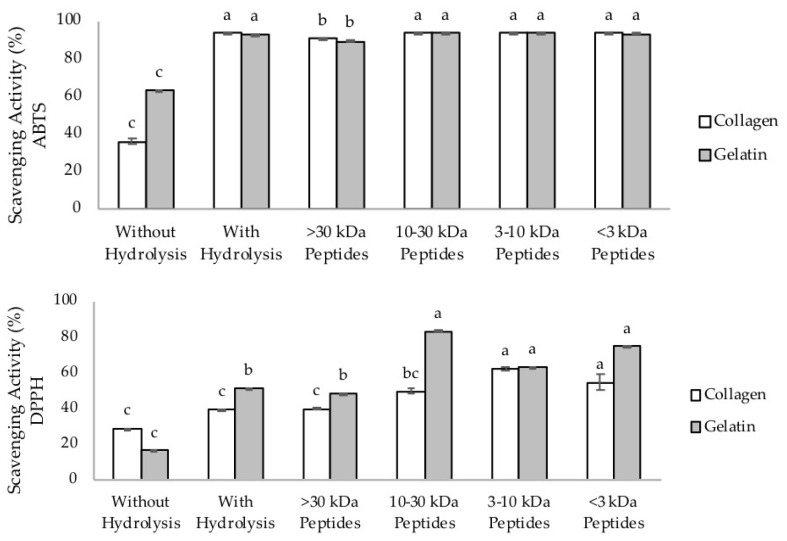
Scavenging activity (in percentage) of tuna skin collagen, gelatin, and hydrolysates. Upper figure, 2,2′-azino-bis (3-ethylbenzothiazoline-6-sulphonic acid (ABTS) method; lower figure, 2,2-diphenyl-1-picrylhydrazyl (DPPH) method. The different letters indicate significant differences (*p* < 0.05).

Scavenging activity of 100% represents complete scavenging of ABTS or DPPH radicals. Hydrolysis processes could increase the scavenging activity for both ABTS and DPPH assays. As shown in Figure 3**,** the scavenging activities of collagen, gelatin, hydrolysate, and the fractions against ABTS were much higher compared with DPPH, as the results represent the scavenging activities against ABTS from tenfold diluted solution in the concentration range of 10–40 µg/mL. The scavenging activities against ABTS radicals of the collagen hydrolysate from milkfish (*Chanos chanos*) skin showed the effectiveness at 1 mg/mL [51]. However, the scavenging activity of nearly 100% was reported for the peptide from horse milk at 1 µg/mL [52]. On the other hand, the gelatin hydrolysate from skipjack scale showed the scavenging activity in the concentration range of 0.1–5.0 mg/mL [8]. The lower the molecular weight, the stronger the scavenging activity tends to become. These data demonstrate that the antioxidant activities of our preparations were satisfactorily high.

In this study, IC_50_ values were classified as follows: Very strong, <0.05 mg/mL; strong, 0.05–0.10 mg/mL; average, 0.10–0.15 mg/mL; weak, 0.15–0.20 mg/mL; and very weak, >0.20 mg/mL. Lower IC_50_ shows higher antioxidant activity [53]. The lower molecular weight peptides tend to show higher antioxidant activities, although variable molecular weight distributions also provide striking differences in the antioxidant properties of hydrolysates [54]. The average molecular weight of protein hydrolysates is one of the most important factors determining biological activity, including the antioxidant one. High antioxidant activity is generally observed for low molecular weight and severed oligopeptides. In addition, the specificity of the protease not only affects the peptide size but also the number and sequence of amino acids in the peptides, which then also affect the antioxidant activity of the hydrolysates [55].

Both ABTS and DPPH methods showed that the highest antioxidant activity of collagen hydrolysate expressed by IC_50_ was obtained for the peptide fractions of 3–10 kDa, and for the gelatin peptide fractions of 10–30 kDa. In this study, the antioxidant activities of gelatin and its hydrolysis were higher than that of collagen. In addition, antioxidant activity of gelatin was found to be higher in the ABTS method. This may be due to the fact that the ABTS assay tends to detect the hydrophobic and hydrophilic compounds, while the DPPH assay is suitable for hydrophobic ones as shown in our previous report [51,56]. In addition, the enzyme Alcalase can produce hydrolysates with higher radical scavenging capacity and iron reducing activity compared to the other enzymes such as collagenase, trypsin, or pepsin [13,45].

All the results obtained in the present study demonstrated that excellent quality of collagen, gelatin, and their hydrolysates with high antioxidant activities can be obtained from the skin waste of the tuna processing industry. These findings will be useful for effective utilization of tuna resources. In addition, these products will be accepted to those communities where the products from livestock sources are rejected due to religious reasons.

## 3. Methods 

### 3.1. Preparation of Collagen 

Collagen was extracted according to the method in our previous report [6]. Pretreatment of tuna skin consisted of two steps, the removal of non-collagenous protein followed by the removal of fat. After cleaning with distilled water, the skin was cut into pieces of approximately 1 × 1 cm. The removal of non-collagenous protein was carried out by immersing the tuna skin 1:10 (*w/v*) in 0.1 M NaOH aqueous solution at 4 °C for 12 h. The NaOH solution was changed every 2 h.

The washed skin was neutralized with distilled water until pH value reached 7, then was immersed in 10% 1:10 (*w/v*) butyl alcohol for 24 h for fat removal, and then neutralized with distilled water. Collagen extraction was carried out with 0.75 M acetic acid at a ratio of 1:10 (*w/v*) at 4 °C for 72 h. Filtration through a filter paper was carried out to collect collagen. The filtered residue was extracted with the same concentration of acetic acid for another 72 h. Subsequently, collagen was precipitated by adding solid NaCl to a final concentration of 1.8 M and subsequent addition of 0.05 M Tris-HCl (pH 7.5) at a ratio of 1:1 (*v/v*) and was allowed to stand for 24 h. The mixture was centrifuged at 3500× g at 4 °C for 1 h. The pellet obtained by the centrifugation was dialyzed against 0.1 M acetic acid, and further against distilled water for 24 h. The obtained collagen was stored frozen at −20 °C.

### 3.2. Preparation of Gelatin 

Gelatin was prepared according to our previously reported method with a slight modification [7]. The extraction was carried out by soaking the cut skin (approximately 1 × 1 cm) with 0.25% citric acid for 12 h at a skin and citric acid ratio of 1:4 (*w/v*). Then, the skin was washed with distilled water and extracted under vigorous agitation at 65 °C for 7 h at a skin and distilled water ratio of 1:1 (*w/v*), followed by filtration through a calico and cotton cloth. The filtrate was dried up with a vacuum evaporator at 60 °C for 50 min.

### 3.3. Preparation of Hydrolysates

The skin collagen and gelatin were hydrolyzed according to Chalamaiah et al. [57] with a slight modification. The collagen and gelatin solution (6.67%, *w/v*) was adjusted to pH 8 with NaOH, and hydrolyzed with Alcalase (2%, *v/v*) (Sigma–Aldrich, St. Louis, MO, USA) at 55 °C for 3 h. The mixture was then left at −20 °C for 5 min for enzyme inactivation. The solution was then centrifuged at 10,000× g at 4 °C for 15 min, and the supernatant was obtained as the hydrolysate.

### 3.4. Fractination

Peptides were fractionated based on their molecular sizes using a molecular weight cut-off (MWCO) membrane 30, 10, and 3 kDa (Millipore Co. Ltd., Waltham, MA, USA) according to Kusumaningtyas et al. [54] with a slight modification. Namely, the peptide solutions were poured into 1.5 mL Eppendorf tubes equipped with a membrane, which were subsequently centrifuged at 5000× g at 22 °C for 10, 15, or 30 min, respectively. The resulting fractions of >30 kDa, 10–30 kDa, 3–10 kDa, and <3 kDa were designated as F1, F2, F3, and F4, respectively.

### 3.5. Molecular Weight Estimation

The protein profile was analyzed based on molecular weight according to Laemmli (1970) by a sodium dodecyl sulfate-polyacrylamide gel electrophoresis (SDS-PAGE) method [58]. For SDS-PAGE analysis, 3% stacking gel and 15% separating gel for collagen and gelatin were used, as well as 17.5% for the hydrolysates. Samples of 2 mg were dissolved in 1 mL of 5% SDS, heated at 85 °C for 1 h, centrifuged at 12,400× g for 5 min. After mixing 20 μL each of the sample and 2× Laemmli buffer, the mixture was heated at 85 °C for 10 min before 15 μL of which was loaded onto the gel well. Electrophoresis was carried out at 13 mA and 100 V for 3 h. Electrophoresis was stopped when the front dye reached about 0.5 cm from the bottom of the gel. Gel staining was carried out using Coomassie brilliant blue, followed by destaining in 25% methanol and 10% acetic acid. The gel was scanned using Photocapt software.

### 3.6. Hydrolysis Degree Analysis

The hydrolysis degree was determined according to Baharrudin [39]. To 20 mL of hydrolysate was added 20 mL of 20% trichloroacetic acid (*w/v*). The mixture was allowed to stand for 30 min, and the precipitate was removed by centrifugation at 6000× g for 30 min. The resulting supernatant was analyzed for nitrogen content by a Kjeldahl method (AOAC 2005). The degree of hydrolysis (DH) was calculated using the following formula:

$$DH\left(\%\right)=\frac{Nitrogen\;content\;in\;the\;supernatant}{Nitrogen\;in\;the\;sample}\times 100$$

### 3.7. Amino Acid Analysis

Amino acid analysis was carried out according to Nollet [59]. Samples of collagen and gelatin (0.1 g) were dissolved in 5 mL of 6 M HCl, vortexed, and then hydrolyzed at 110 °C for 22 h. The hydrolyzed sample was transferred to a 50 mL measuring flask and set up to the boundary mark. The sample was filtered through a 0.45 μm nitrocellulose filter (ThermoFischer Scientific, Waltham, MA, USA), and 500 μL of the filtrate was mixed with 40 μL of α-aminobutyric acid (AABA) and 460 μL of Aquabidest. To the mixture of 10 μL was added 70 μL of AccQ-Fluor Borate, and it was then vortexed. To the homogenate was added 20 μL of fluorine reagent and it was then incubated at 55 °C for 10 min. The prepared samples were analyzed with the Ultra Performance Liquid Chromatography (UPLC) system (Shimadzu, Tokyo, Japan).

The standard solution analysis was carried out by mixing 40 μL of amino acid standards with an equal volume of the internal standard (AABA) and 920 μL of distilled water, and then homogenized. The standard of 10 μL was pipetted and mixed with 70 μL AccQ-Fluor Borate, and then vortexed. To the homogenate was added 20 μL of fluorine reagent and it was incubated at 55 °C for 10 min. The prepared samples were analyzed as described above.

### 3.8. Antioxidant Quantification

The antioxidant activity was assayed according to ABTS and DPPH methods [51]. For the ABTS assay, a total of 100 μL sample solution was mixed with 200 μL ABTS solution in the microplate wells and placed at room temperature for 10 min. The absorbance was measured at 405 nm. For the DPPH assay, 0.2 mM DPPH radical was added to 96% ethanol. DPPH solution was then measured at 540 nm until an absorbance of 1.1 ± 0.05 was obtained. To a total of 100 μL sample solution was added 200 μL of DPPH, which was then allowed to stand for 30 min. The absorbance of the mixture was measured at 540 nm. The scavenging activity of peptide fractions to ABTS and DPPH radicals was expressed using equation:

$$Scavenging\;activity\;\left(\%\right)=\frac{\left(A0-A1\right)}{A0}\times 100$$

where

*A*0 = absorbance of ABTS/DPPH, and
*A*1 = final absorbance.

Inhibition concentration of 50% free radical activity (IC_50_) values were calculated using the linear regression equation. IC_50_ values were obtained by entering y = 50 and the known values a and b. The value of x as IC50 can be calculated by the following equation:

y=bx+a

where

y = antioxidant activity,
x = sample concentration,
a = slope,
b = intercept.

The concentration of the sample and antioxidant activity were plotted on the x and y axes, respectively, in the linear regression equation. The linear regression equation obtained in the form of the equation y = bx + a was used to find the value of IC_50_ of each sample by stating a value of y (50) and the value of x to be obtained as IC_50_. The IC_50_ value states the concentration of the sample solution needed to reduce free radicals by 50%.

### 3.9. Statistical Analysis

The quantitative data of the test results were processed using Excel 2013 (Microsoft, Redmond, WA, USA), Minitab 162013 (Minitab, Sate College, PA, USA), and SPSS Statistics 222013 (IBM, Armonk, NY, USA). Data were analyzed descriptively, while the experiments were designed using completely randomized design data for antioxidant activities. Data analysis was performed with one-way analysis of variance (ANOVA) at a 95% confidence interval (α = 0.05). The level that could affect the response was further checked using a Tukey test.

## 4. Conclusions

Collagen and gelatin could be successfully prepared from tuna skin waste, which is abundantly available from tuna processing. Both the collagen and the gelatin were found to be of high quality based on the standards for commercial use. The derived peptides from both the collagen and gelatin showed strong antioxidant activities, mainly in the ABTS assay as demonstrated by the scavenging activity and IC_50_ values. The highest antioxidant activity (lowest IC_50_) of collagen hydrolysate was obtained for the peptide fractions of 3–10 kDa and for the gelatin peptide fractions of 10–30 kDa. Those of the hydrolysates and the derived peptides of gelatin were categorized as very strong. Tuna skin waste is thus considered to be an excellent source of antioxidant peptides, making it possible to effectively utilize the waste and add value to tuna resources. Further study is now ongoing to identify the strong antioxidant components obtained by high performance liquid chromatography. 

## Figures and Tables

**Table 1:** Characteristics of tuna skin collagen and gelatin.

Parameter	Collagen	Gelatin
Yield (%)	22.63 ± 2.04	19.97 ± 0.79
pH	6.63 ± 0.03	4.94 ± 0.01
Color		
L* (lightness)	97.57 ± 0.94	56.46 ± 0.78
a* (redness)	0.93 ± 0.75	2.56 ± 0.55
b* (yellowness)	1.21 ± 1.17	22.38 ± 0.38
Whiteness (%)	96.69 ± 0.35	50.97 ± 0.72

**Table 2:** Hydrolysis degree (HD) of tuna skin collagen and gelatin by 2% (*v/v*) Alcalase hydrolysis for three hours.

Sample	Hydrolysis Degree (%)
Collagen	52.71 ± 1.54
Gelatin	45.29 ± 0.01

**Table 3:** Amino acid composition of tuna skin collagen, gelatin, and hydrolysates (g/100 g).

Amino Acid	Collagen	Collagen Hydrolysate	Gelatin	Gelatin Hydrolysate
Arginine	3.48	3.27	9.16	8.48
Lysine	2.24	1.78	4.21	4.86
Threonine	1.48	1.41	3.40	3.38
Phenylalanine	0.95	0.89	2.68	2.29
Leucine	1.13	1.04	2.68	2.63
Valine	0.96	0.96	2.22	2.27
Isoleucine	0.48	0.44	1.17	1.15
Histidine	0.31	0.29	0.85	0.72
Glycine	10.44	8.85	25.98	24.45
Proline	5.10	4.09	11.94	11.86
Alanine	5.03	3.64	10.50	11.20
Glutamate	5.05	3.34	10.31	11.61
Aspartate	2.73	1.93	5.12	5.83
Serine	1.53	1.35	3.78	3.67
Tyrosine	0.21	0.17	0.59	0.54

**Table 4:** Antioxidant activities of tuna skin collagen, gelatin, and their hydrolysates.

Samples	IC_50_ (µg protein/mL)	
	ABTS	DPPH
Collagen	313.29 ± 0.15 ^f^	560.51 ± 0.02 ^g^
Hydrolyzed collagen	66.28 ± 0.12 ^e^	119.10 ± 0.01 ^f^
Peptides >30 kDa	64.47 ± 0.13 ^e^	101.77 ± 0.01 ^f^
Peptides 10–30 kDa	33.02 ± 0.05 ^cd^	83.22 ± 0.01 ^de^
Peptides 3–10 kDa	29.24 ± 0.02 ^c^	75.94 ± 0.01 ^d^
Peptides <3 kDa	35.39 ± 0.07 ^d^	82.12 ± 0.02 ^de^
Gelatin	62.21 ± 0.01 ^e^	654.62 ± 0.03 ^g^
Hydrolyzed gelatin	16.28 ± 0.01 ^b^	78.87 ± 0.01 ^d^
Peptides >30 kDa	19.17 ± 0.01 ^b^	84.15 ± 0.01 ^e^
Peptides 10–30 kDa	9.11 ± 0.01 ^a^	15.12 ± 0.01 ^a^
Peptides 3–10 kDa	17.06 ± 0.01 ^b^	50.04 ± 0.01 ^c^
Peptides <3 kDa	11.20 ± 0.01 ^ab^	23.80 ± 0.01 ^b^

Note: Different superscripts in the same column show significant differences (*p* < 0.05).
